# Correlation between ultrasound-diagnosed non-alcoholic fatty liver and periodontal condition in a cross-sectional study in Japan

**DOI:** 10.1038/s41598-018-25857-z

**Published:** 2018-05-14

**Authors:** Takahiro Iwasaki, Akiko Hirose, Tetsuji Azuma, Tamie Ohashi, Kazutoshi Watanabe, Akihiro Obora, Fumiko Deguchi, Takao Kojima, Atsunori Isozaki, Takaaki Tomofuji

**Affiliations:** 1 Department of Community Oral Health, School of Dentistry, Asahi University, 1851 Hozumi, Mizuho, Gifu, 501-0296 Japan; 2 Asahi University Hospital, 3-23 Hashimoto-cho, Gifu, Gifu, 500-8523 Japan; 3 School of Dental Hygienists, Asahi University, 1851 Hozumi, Mizuho, Gifu, 501-0296 Japan

## Abstract

This cross-sectional study investigated the relationship between periodontal condition and ultrasound-diagnosed non-alcoholic fatty liver disease (NAFLD) in a Japanese oral health check population. A total of 1226 consecutive participant were enrolled in the study. Abdominal ultrasonography was applied to diagnose NAFLD. Of the study participants, 339 (27.7%) had ultrasonography-diagnosed NAFLD. The participants with NAFLD had a significantly higher prevalence of probing pocket depth (PPD) ≥ 4 mm (86.7%) than those without NAFLD (72.9%) (*p* < 0.05). After adjusting for gender, age, Brinkman index, regular exercise habits, body mass index, number of teeth present, presence of periodontitis, blood pressure, and serum parameters, there was a statistically significant difference in the adjusted odds ratios of having PPD ≥ 4 mm for NAFLD (Odds ratio = 1.881, 95% confidence interval 1.184–2.987, *p* < 0.01). Having PPD ≥ 4 mm may be a risk factor for ultrasound-diagnosed NAFLD in this cross-sectional study of a Japanese oral health check population.

## Introduction

Non-alcoholic fatty liver disease (NAFLD) is increasing recognized as one of the most common chronic liver diseases^1^. NAFLD encompasses a spectrum of diseases from simple hepatic steatosis to non-alcoholic steatohepatitis. While simple steatosis represents a relatively small health issue, steatohepatitis is of significant concern as it can potentially progress to liver cirrhosis and hepatocellular carcinoma^2^. The prevalence of NAFLD in Japanese adults is about 30%^3,4^. Although the mechanism of NAFLD is unknown, it can occur in association with metabolic diseases, such as obesity^5^, type 2 diabetes mellitus^6^, hypertension^7^ and hyperlipidemia^8^.

Periodontal disease is a chronic inflammatory disease of the supporting structures of the teeth. Increasing evidence indicates that periodontal disease is associated with many metabolic diseases, such as diabetes mellitus^9^ and cardiovascular disease^10^. Thus, since NAFLD is a metabolic disease, periodontal disease may be also associated with NAFLD. In animal studies, experimental periodontal disease induces increased blood levels of inflammatory molecules and oxidative stress, contributing to hepatic steatosis^11^. We also found that the improvement of periodontal inflammation reduced hepatic steatosis following periodontitis^12^. These observations support the hypothesis that the presence of periodontal disease may be a risk factor for NAFLD.

Clinical investigations have focused on the relationship between NAFLD and periodontal disease. It was reported that periodontal disease is more common in NAFLD patients with significant fibrosis compared to those with mild or no fibrosis^13^. It was also shown that relative to participants lacking clinical attachment level (CAL) ≥3 mm, the incidence of NAFLD was slightly elevated in participants with <30% of sites affected and moderately elevated in participants with ≥30% of sites affected, respectively^14^. However, since very little information is available about the relationship between NAFLD and periodontal disease in humans, additional clinical works are needed. In Japan, health check-ups in the hospital, including oral examinations, are popular. In addition, it is accepted that having probing pocket depth (PPD) ≥4 mm indicates that the individual has periodontal disease^15^. Therefore, the purpose of this cross-sectional study was to investigate the relationship between NAFLD and having PPD ≥4 mm in a Japanese oral health check population.

## Results

Table 1 presents the characteristics of the participants. The overall prevalence of NAFLD was 27.7%. There were significant differences between the participants with and without NAFLD with respect to gender, age, body mass index (BMI), waist circumference (WC), Brinkman index, and having PPD ≥ 4 mm (*p* < 0.001). There were also significant differences between the participants with and without NAFLD with respect to serum aspartate aminotransferase (AST), alanine aminotransferase (ALT), γ-glutamyl transferase (GGT), systolic blood pressure (SBP), diastolic blood pressure (DBP), hemoglobin A1c (HbA1c), triglyceride, high-density lipoprotein (HDL) cholesterol, low-density lipoprotein (LDL) cholesterol, and C-reactive protein (CRP) concentrations (*p* < 0.001).

**Table 1:** Comparison of Characteristics between Subjects with and without NAFLD.

	All (n = 1226)	Without NAFLD (n = 887)	With NAFLD (n = 339)	*P* value^†^
Gender, (M/F)(%)^*^	772/454 (63.0%/37.0%)	491/396 (55.4%/44.6%)	281/58 (82.9%/17.1%)	<0.001
Age, years	50 (41, 58)	48 (40, 57)	53 (44, 59)	<0.001
BMI, kg/m^2^	22.4 (20.4, 24.7)	21.4 (19.6, 23.2)	25.5 (23.4, 27.2)	<0.001
WC, cm	79.0 (73.0, 85.0)	76.0 (70.0, 81.5)	87.0 (82.0, 92.0)	<0.001
Brinkman index	0.0 (0.0, 288.5)	0.0 (0.0, 200.0)	140.0 (0.0, 500.0)	<0.001
Present teeth (n)	28 (27, 29)	28 (27, 29)	28 (27, 29)	0.143
BOP, (Absence/Presence)(%)^*^	112/1114 (9.1%/90.9%)	83/804 (9.4%/90.6%)	29/310 (8.6%/91.4%)	0.740
Periodontitis, (PPD ≤ 3 mm/PPD ≥ 4 mm)(%)^*^	285/941 (23.2%/76.8%)	240/647 (27.1%/72.9%)	45/294 (13.3%/86.7%)	<0.001
Regular exercise habits, (Apply/Not apply)^*^	236/990 (19.2%/80.8%)	180/707 (20.3%/79.7%)	56/283 (16.5%/83.5%)	0.145
AST, U/L	16.0 (13.0, 20.0)	15.0 (12.0, 19.0)	20.0 (15.0, 26.0)	<0.001
ALT, U/L	16.0 (12.0, 22.0)	14.0 (11.0, 18.0)	25.0 (18.0, 36.0)	<0.001
GGT, U/L	18.0 (13.0, 28.0)	15.0 (12.0, 22.0)	27.0 (19.0, 42.0)	<0.001
SBP, mmHg	117.0 (107.0, 128.0)	114.0 (105.0, 125.0)	125.0 (116.0, 134.0)	<0.001
DBP, mmHg	72.0 (64.8, 80.0)	69.0 (62.0, 77.0)	78.0 (71.0, 85.0)	<0.001
HbA1c, %	5.4 (5.3, 5.6)	5.4 (5.2, 5.6)	5.6 (5.4, 5.9)	<0.001
Total cholesterol, mg/dL	202.0 (180.0, 223.0)	200.0 (179.0, 222.0)	205.0 (181.0, 226.0)	0.117
Triglyceride, mg/dL	54.5 (46.0, 97.0)	57.0 (41.0, 80.0)	97.0 (68.0, 135.0)	<0.001
HDL, mg/dL	63.0 (51.0, 76.0)	67.0 (57.0, 79.0)	52.0 (45.0, 61.0)	<0.001
LDL, mg/dL	110.0 (93.0, 129.0)	107.0 (92.0, 125.0)	121.0 (100.0, 137.0)	<0.001
CRP, mg/dL	0.04 (0.01, 0.08)	0.03 (0.01, 0.06)	0.07 (0.04, 0.16)	<0.001

Continuous variables are expressed as median (first quartile, third quartile) deviation.

^*^n (%); ^†^Chi-square test (Direct method of Fisher) or Mann Whitney U test.

Abbreviations: NALFD, non-alcoholic fatty liver disease; M, male; F, Female; BMI, body mass index; WC, waist circumference; PPD, probing pocket depth; BOP, bleeding on probing; AST, aspartate aminotransferase; ALT, alanine aminotransferase; GGT, γ-glutamyltransferase; SBP, systolic blood pressure; DBP, diastolic blood pressure; HbA1c, hemoglobin A1c; HDL, high-density lipoprotein; LDL, low-density lipoprotein; CRP, C-reactive protein.

**Figure 1:**
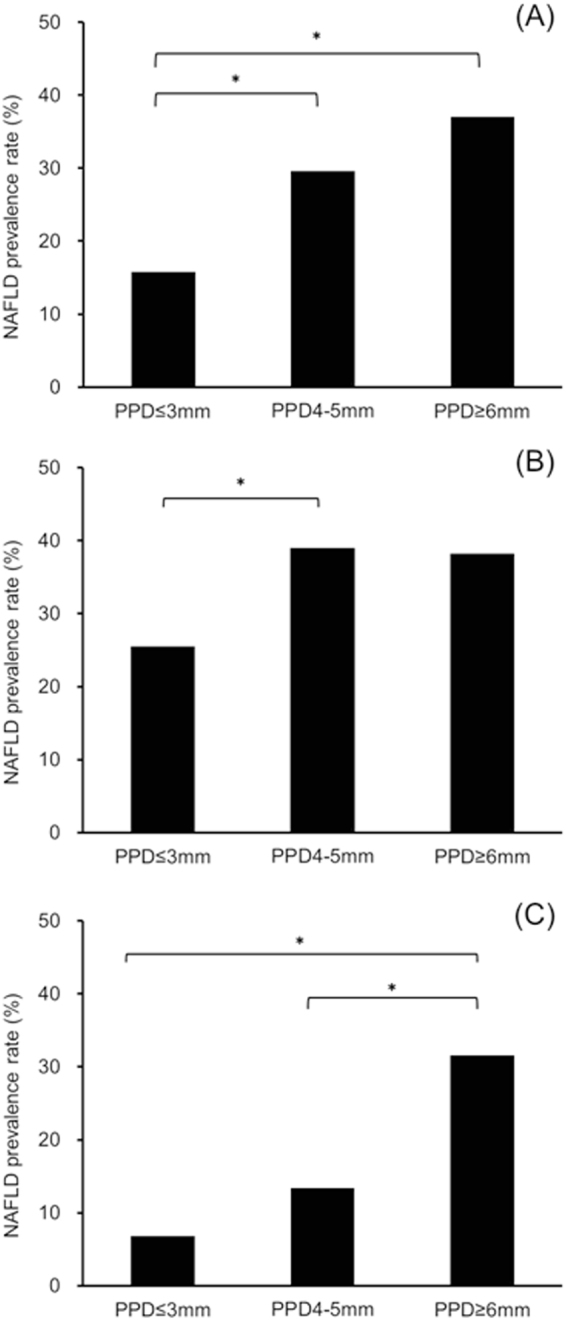
Differences in prevalence rate of non-alcoholic fatty liver disease according to periodontal condition in all (**A**), male (**B**), and female (**C**) participants. NAFLD, non-alcoholic fatty liver disease and PPD, probing pocket depth. *p < 0.05, compared with the participants with PPD ≤ 3 mm, using the Kruskal Wallis test with post hoc Mann Whitney U test (corrected Bonferroni’s method).

Comparative results of the participants with different severity of periodontal disease are shown in Fig. 1. In all participants and the female participants, the prevalence rate of NAFLD increased according to the severity of periodontal disease. There were significant differences between the participants with PPD ≤ 3 mm and PPD = 4–5 mm in the prevalence rate of NAFLD in male participants (*p* < 0.05). In all participants, there were significant differences between the participants with PPD ≤ 3 mm and PPD = 4–5 mm (*p* < 0.001), PPD ≤ 3 mm and PPD ≥ 6 mm (*p* < 0.001). In females, there were significant differences between the participants with PPD = 4–5 mm and PPD ≥ 6 mm (*p* < 0.001), PPD ≤ 3 mm and PPD ≥ 6 mm (*p* < 0.05).

Table 2 shows the results of the logistic regression analysis with prevalence of NAFLD as the dependent variable in all participants. The prevalence of NAFLD was related to gender (female, odds ratio (OR) = 0.583, *p* < 0.05), age (OR = 1.024, *p* < 0.05), BMI (OR = 1.470, *p* < 0.001), having PPD ≥ 4 mm (OR = 1.881, *p* < 0.01), HbA1c level (OR = 1.594, *p* < 0.05), total cholesterol concentration (OR = 0.968, *p* < 0.05), LDL cholesterol concentration (OR = 1.040, *p* < 0.01), triglyceride concentration (OR = 1.011, *p* < 0.001), DBP (OR = 1.050, *p* < 0.01), and CRP concentration (OR = 3.208, *p* < 0.01) after adjusting for gender, age, Brinkman index, regular exercise habits, BMI, number of present teeth, having PPD ≥ 4 mm, HbA1c level, total cholesterol concentration, HDL cholesterol concentration, LDL cholesterol concentration, triglyceride concentration, SBP, DBP, and CRP concentration.

**Table 2:** Factor associated with NAFLD in Study Populations by Multivariate Logistic Regression Analysis.

Covariate	Odds Ratio	95% Confidence level	*P* value
Gender, (F)	0.583	0.368–0.923	0.021
Age, years	1.024	1.005–1.044	0.014
Brinkman index	1.000	0.999–1.000	0.244
Regular exercise habits (Not apply)	1.349	0.878–2.072	0.172
BMI	1.470	1.373–1.575	<0.001
Present teeth (n)	0.998	0.947–1.052	0.943
Periodontitis, (PPD ≥ 4 mm)	1.881	1.184–2.987	0.007
HbA1c, %	1.594	1.089–2.333	0.016
Total cholesterol, mg/dL	0.968	0.943–0.994	0.017
Triglyceride, mg/dL	1.011	1.006–1.017	<0.001
HDL, mg/dL	1.010	0.980–1.040	0.524
LDL, mg/dL	1.040	1.010–1.072	0.008
SBP, mmHg	0.980	0.958–1.001	0.067
DBP, mmHg	1.050	1.020–1.080	0.001
CRP, mg/dL	3.208	1.625–6.330	0.001

Abbreviations: NALFD, non-alcoholic fatty liver disease; F, Female; BMI, body mass index; WC, waist circumference; PPD, probing pocket depth; HbA1c, hemoglobin A1c; HDL, high-density lipoprotein; LDL, low-density lipoprotein; SBP, systolic blood pressure; DBP, diastolic blood pressure; CRP, C-reactive protein.

Table 3 presents the results of the logistic regression analysis with prevalence of NAFLD as the dependent variable in male and female participants. In male participants, the prevalence of NAFLD was related to BMI (OR = 1.476, *p* < 0.001), triglyceride concentration (OR = 1.010, *p* < 0.01), SBP (OR = 0.969, *p* < 0.05), DBP (OR = 1.061, *p* < 0.01), and CRP concentration (OR = 2.757, *p* < 0.01). In female participants, the prevalence of NAFLD was related to age (OR = 1.067, *p* < 0.05), BMI (OR = 1.472, *p* < 0.001), having PPD ≥ 4 mm (OR = 2.972, *p* < 0.05), total cholesterol concentration (OR = 0.902, *p* < 0.01), triglyceride concentration (OR = 1.025, *p* < 0.01), HDL cholesterol concentration (OR = 1.103, *p* < 0.05), and CRP concentration (OR = 8.736, *p* < 0.05).

**Table 3:** Factor associated with NAFLD in Different Populations in Gender by Multivariate Logistic Regression Analysis.

Covariate	Odds Ratio	95% Confidence level	*P* value
**Male**			
Age, years	1.016	0.995–1.038	0.130
Brinkman index	1.000	0.999–1.000	0.295
Regular exercise habits	1.272	0.792–2.042	0.319
BMI	1.476	1.354–1.608	<0.001
Present teeth (n)	0.973	0.918–1.032	0.363
Periodontitis, (PPD ≥ 4 mm)	1.620	0.945–2.777	0.080
HbA1c, %	1.498	0.997–2.251	0.052
Total cholesterol, mg/dL	0.976	0.948–1.005	0.109
Triglyceride, mg/dL	1.010	1.003–1.016	0.003
HDL, mg/dL	0.995	0.963–1.028	0.777
LDL, mg/dL	1.029	0.996–1.063	0.087
SBP, mmHg	0.969	0.944–0.994	0.016
DBP, mmHg	1.061	1.025–1.097	0.001
CRP, mg/dL	2.757	1.399–5.432	0.003
**Female**			
Age, years	1.067	1.012–1.125	0.017
Brinkman index	1.001	0.998–1.005	0.486
Regular exercise habits	2.062	0.684–6.212	0.199
BMI	1.472	1.291–1.679	<0.001
Present teeth (n)	1.121	0.991–1.268	0.068
Periodontitis, (PPD ≥ 4 mm)	2.972	1.107–7.979	0.031
HbA1c, %	2.030	0.672–6.128	0.209
Total cholesterol, mg/dL	0.902	0.836–0.974	0.008
Triglyceride, mg/dL	1.025	1.008–1.043	0.004
HDL, mg/dL	1.103	1.019–1.195	0.016
LDL, mg/dL	1.124	1.035–1.222	0.060
SBP, mmHg	1.013	0.965–1.063	0.596
DBP, mmHg	1.028	0.966–1.095	0.386
CRP, mg/dL	8.736	1.009–75.606	0.049

Abbreviations: NALFD, non-alcoholic fatty liver disease; F, Female; BMI, body mass index; WC, waist circumference; PPD, probing pocket depth; HbA1c, hemoglobin A1c; HDL, high-density lipoprotein; LDL, low-density lipoprotein; SBP, systolic blood pressure; DBP, diastolic blood pressure; CRP, C-reactive protein.

There were significant differences in serum HbA1c level between the participants with PPD ≤ 3 mm and PPD ≥ 6 mm (*p* < 0.01) and those with PPD = 4–5 mm and PPD ≥ 6 mm (*p* < 0.05) (Table 4). There were also significant differences in serum CRP concentration between the participants with PPD ≤ 3 mm and PPD ≥ 6 mm (*p* < 0.01) and those with PPD = 4–5 mm and PPD ≥ 6 mm (*p* < 0.05).

**Table 4:** Comparisons of HbA1c and CRP in different periodontal condition.

	PPD ≤ 3 mm (n = 285)	PPD = 4-5 mm (n = 730)	PPD ≥ 6 mm (n = 211)
HbA1c, %	5.4 (5.3, 5.6)	5.4 (5.3, 5.6)	5.5 (5.3, 5.8) ^* †^
CRP, mg/dL	0.04 (0.01, 0.07)	0.04 (0.01, 0.07)	0.06 (0.03, 0.14) ^* †^

Continuous variables are expressed as median (first quartile, third quartile) deviation.

^*^p < 0.01, compared with the participants with PPD ≤ 3 mm, using the Kruskal Wallis test with post hoc Mann Whitney U test (corrected Bonferroni’s method).

^†^*p* < 0.05,compared with the participants with PPD = 4–5 mm, using the Kruskal Wallis test with post hoc Mann Whitney U test (corrected Bonferroni’s method).

Abbreviations: PPD, probing pocket depth; HbA1c, hemoglobin A1c; CRP, C-reactive protein.

## Discussion

This cross-sectional study assessed the relationship between NAFLD and periodontal condition in a Japanese oral health check population. We found that the group with NAFLD had a higher prevalence of having PPD ≥ 4 mm than that without NAFLD. In addition, the group with PPD ≥ 4 mm had higher risk of NAFLD than the group without PPD ≥ 4 mm after adjusting for gender, age, Brinkman index, regular exercise habits, BMI, number of teeth present, presence of having PPD ≥ 4 mm, HbA1c level, total cholesterol concentration, triglyceride concentration, HDL cholesterol concentration, LDL cholesterol concentration, SBP, DBP, and CRP concentration. This indicates that the presence of periodontal disease may increase the risk of NAFLD in the present population.

Our logistic regression analysis also showed that the presence of periodontitis was associated with NAFLD in female participants, but not in male participants. This suggests that there is a sex difference in the association between NAFLD and periodontal disease. In this study, the prevalence rate of having PPD ≥ 4 mm was 82.3% in male participants and 67.4% in female participants. Most of the male participants had PPD ≥ 4 mm, which may represent a bias that reduced the influence of periodontal disease on NAFLD.

In the present study, the increases in BMI, DBP and serum parameters, including triglyceride, LDL cholesterol, and HbA1c, were associated with NAFLD risk. These observations suggest that obesity, hypertension, hyperlipidemia, and type 2 diabetes mellitus could increase the risk for NAFLD. These are consistent with previous reports that demonstrated a positive relationship between NAFLD and other metabolic diseases^16–18^. In addition, we found that the increase in serum CRP concentration was also associated with NAFLD risk. This is in agreement with the previous findings, which showed that a 1 mg/dL increase in high sensitivity CRP level increased the risk of developing NAFLD by 1.7 fold as compared to control^16^.

In our findings, the serum HbA1c level was higher in the participants with PPD ≥ 6 mm than those with PPD ≤ 3 mm. This indicates that periodontitis induced an elevation in serum HbA1c level. Investigators have reported that increased serum HbA1c level is an independent risk factor for NAFLD^17,18^. It is feasible that periodontal disease could be detrimental to hepatic health through increased serum HbA1c level.

Animal studies have suggested that the increased serum level of inflammatory cytokines following periodontal disease contributed to NAFLD progression^11^. A clinical study also showed that the relationship between NAFLD and periodontal disease was modified by serum CRP concentration^19^. In the present study, the results showed that serum CRP concentration tended to increase according to the severity of periodontal disease. In particular, serum CRP concentration was significantly higher in the participants with PPD ≥ 6 mm than those with PPD ≤ 3 mm. This suggests that circulating inflammatory molecules play a crucial role in the association between NAFLD and periodontal disease. However, not only periodontal inflammation but also the inflammation of NAFLD could contribute to the elevation of serum CRP concentration. Additional studies are needed to clarify this point.

In our findings, the prevalence rate of NAFLD in male was higher in the participants with PPD 4–5 mm than those with PPD ≤ 3 mm, while that in female was higher in the participants with PPD ≥ 6 mm than those with PPD ≤ 3 mm or PPD 4–5 mm. The results indicate gender differences in the association between NAFLD and periodontal condition. This is consistent with the previous study, which revealed that gender differences seem to exist in the association between periodontal disease and metabolic syndrome^20^. It is known that sex hormones play an important role in the process of both periodontal inflammation^21^ and NAFLD^22^. The reason for the gender differences in the association between NAFLD and periodontal condition may appear due to sex hormones.

The gold standard diagnostic test for NAFLD is liver biopsy. However, since it is not reasonable to use the highly invasive liver biopsy as a diagnostic test in a health-check population, ultrasonography was used to detect NAFLD in this study. A meta-analysis shows that the overall sensitivity and specificity of ultrasound for detection of moderate-severe fatty liver compared to histology (the gold standard) were 84.8% and 93.6%, respectively^23^. This meta-analysis also revealed that the summary area under the receiver operating characteristics curve was 0.93. Therefore, it is suggested that ultrasound is an accurate, reliable imaging technique for the detection of NAFLD.

An epidemiological study demonstrated that periodontitis was significantly more common in patients with biopsy-proven non-alcoholic steatohepatitis and any fibrosis than without non-alcoholic steatohepatitis^13^. Another clinical study suggested that infection with the periodontal pathogenic bacteria *Aggregatibacter actinomycetemcomitans* affects NAFLD by altering the gut microbiota and glucose metabolism^24^. Furthermore, a cohort investigation clarified that relative to participants lacking CAL ≥3 mm, NAFLD incidence was elevated slightly in participants with <30% of CAL sites affected and moderately in participants with ≥30% of CAL sites affected^14^. These observations are consistent with the present concept that periodontal disease could increase the risk of NAFLD.

Increasing evidence has shown that periodontal disease may be associated with multiple metabolic diseases, such as diabetes mellitus ^9^, cardiovascular disease^10^, and atherosclerosis^25^. The present results have clarified that periodontal disease may be linked to NAFLD. In Japan, the Industrial Safety and Health Act stipulates that Japanese companies must offer annual health examinations for all employees in order to prevent metabolic diseases. However, the oral health examination is optional. The present and previous studies indicate the importance of periodontal examination in order to assess the risk of metabolic diseases in the health-check population.

This study has some limitations. First, all participants were recruited at the Asahi University Hospital. This may limit the ability to extrapolate our findings to the general population. Additionally, the present study was a cross-sectional study, and hence cannot demonstrate a causal relationship. Additional longitudinal studies are needed to investigate the relationship between NAFLD and having PPD ≥ 4 mm. Furthermore, it might be important to confirm the severity of NAFLD by liver biopsy, because the severity of NAFLD itself would affect the relationship between periodontal condition and NAFLD. On the other hand, the strength of this study is the sufficient sample size needed to assess the prevalence of NAFLD in participants with PPD ≥ 4 mm.

In conclusion, there appears to be a positive association between ultrasound-diagnosed NAFLD and having PPD ≥ 4 mm in a cross-sectional study in Japan.

## Methods

### Study population

The participants of this study consisted of 1280 Japanese who underwent oral health check-ups from Jan 2016 through Dec 2016 at the Asahi University Hospital in Gifu, Japan. Because the present study involves completing a survey, it was not necessary to perform sample size calculations. We excluded 37 participants with insufficient data. In addition, participants who had chronic hepatitis C infection (n = 6) and chronic hepatitis B infection (n = 11) were also excluded. In addition, because no participants reported alcohol intake of ≥20 g/day, we did not exclude participants who consumed alcohol^26^. Furthermore, there was no participant with the autoimmune hepatic disease. Accordingly, 1226 participants (772 men, 454 women) were eligible for this study. The study protocol was approved by the Ethics Committee of Asahi University (No. 27010). The study was performed in accordance with the Declaration of Helsinki. All participants provided written informed consent prior to study participation.

### Diagnosis of fatty liver

NAFLD was defined as fatty liver detected by ultrasonography (ProSound Alpha 7, Hitati Aloka Medical, Tokyo, Japan) in the absence of other causes of chronic liver disease (i.e., hepatitis C antibody-negative, hepatitis B surface antigen-negative, alcohol consumption <20 g/day)^26^. An ultrasonographical diagnosis of fatty liver was defined as a bright liver, increased liver echotexture compared with kidneys, vascular blurring, and deep attenuation of the liver. This diagnosis was performed by two specialists in internal medicine.

### Measurement of biochemical markers

Venous blood samples were collected after an overnight fast. Chemiluminescence immunoassay (ARCHITECT HBsAg QT / ARCHITECT HCV, ABBOTT JAPAN, Tokyo, Japan) was used to test serum HBV surface antigen and antibody to HCV. The simultaneous multi-item automatic analyser (Dimension Vista 1500, Siemens Healthineers Japan, Toyko, Japan) was utilized to determine serum biochemical markers, including AST, ALT, GGT, total cholesterol, triglyceride, HDL cholesterol, LDL cholesterol, and CRP. In addition, the diabetes item automatic analyser (DM-JACK, Kyowa Medex, Tokyo, Japan) was utilized to determine HbA1c.

### Assessment of body composition

An automatic height scale with body composition meter (TBF-110/TBF-210/DC-250, TANITA, Tokyo, Japan) was used to measure participants’ height and body weight. WC was measured by a nurse. BMI was computed as weight in kilograms divided by the square of height in meters.

### Measurement of blood pressure

An automatic blood pressure monitor (HBP-9021/HBP-9020/BP-230RV3, OMRON HEALTHCARE, Kyoto, Japan) was used to measure SBP and DBP.

### Oral examination

Three dentists examined the oral health status of the study participants. The number of teeth in the mouth was counted. PPD was assessed using a periodontal probe (YDM, Tokyo, Japan) at six sites (mesio-buccal, mid-buccal, disto-buccal, disto-lingual, mid-lingual and mesio-lingual) per tooth. The presence or absence of teeth exhibiting bleeding on probing (BOP) was recorded. Good intra- and inter-examiner agreement was achieved for repeated PPD measurements (Kappa statistic, >0.8).

### Questionnaire

Participants were asked to complete a questionnaire regarding their health behaviors. The questionnaire included the following items: age, sex, presence or absence of regular exercise habit, alcohol habit, history of hepatic disease and smoking status (Brinkman index).

### Statistical analysis

In this study, one or more teeth with ≥4 mm PPD was defined as the presence of periodontitis^27^. A chi-square test and the Mann-Whitney *U* test were used to assess significant differences in selected characteristics between study participants with and without NAFLD. The Kruskal-Wallis test with the post hoc Mann-Whitney *U* test (corrected Bonferroni’s method) was used for three group comparisons with different severity of periodontal disease (all teeth with PPD ≤ 3 mm, one or more teeth with PPD 4–5 mm, or one or more teeth with PPD ≥ 6 mm). Logistic regression analyses were also performed with the presence or absence of NAFLD as dependent variables. Independent variables were selected when the *p* value was <0.20 for the chi-square test and the Mann-Whitney *U* test in each variable, since previous studies have suggested that potential confounders should be eliminated only if *p* > 0.20, in order to prevent residual confounding^28^.

Analyses were performed using a statistical package (IBM SPSS statistics version 24, IBM Japan, Tokyo, Japan). All reported *p* values were considered statistically significant if less than 0.05.
